# A Cadaveric Case of Palmaris Longus Agenesis and Reversal

**DOI:** 10.7759/cureus.47664

**Published:** 2023-10-25

**Authors:** Christopher Pham, Peter Vien, Abigail Hielscher

**Affiliations:** 1 Department of Medicine, The Robert Larner, M.D. College of Medicine, University of Vermont, Burlington, USA; 2 Department of Neurological Sciences, The Robert Larner, M.D. College of Medicine, University of Vermont, Burlington, USA

**Keywords:** bilateral variation, reversal, anatomical variation, agenesis, cadaveric dissection, muscle variation, palmaris longus tendon

## Abstract

The palmaris longus frequently exhibits anatomical variations with palmaris longus agenesis and reversal being the most prevalent. These variations are relevant clinically, as the muscle is often used during plastic surgeries for grafting tendons. They are also relevant in pathology, as hypertrophy of the reversed muscle is related to median nerve compression. In this report, we describe an unusual case in which a male cadaver-donor exhibited a right-sided palmaris longus reversal and left-sided palmaris longus agenesis. Review of the literature documents no previous co-occurrence of these anomalies. Since the muscle has relevance in a variety of contexts within medicine and surgery, reporting on a variation like this carries significant educational and clinical value.

## Introduction

The palmaris longus (PL) is a highly variable structure in the human body. This thin muscle is one of the superficial flexor muscles of the forearm, innervated by the median nerve and supplied by the ulnar artery. Anatomically, it is located medial to the flexor carpi radialis and lateral to the flexor carpi ulnaris (FCU), originating from the medial epicondyle of the humerus. It becomes tendinous in the middle third of the forearm and inserts at the palmar aponeurosis in the wrist. 

The function of PL is debated. When present, it acts as a weak flexor of the wrist and tenses the palmar aponeurosis. However, there is no dysfunction or pathology in humans associated with its absence [1]. Presence of PL varies greatly among populations, with absence rates ranging from 1.5% to 63.9% depending on origin [2,3]. Absence can be unilateral or bilateral, with unilateral absence of the left PL being more common [2,4]. Other variations include muscle belly duplication (two muscle bellies with the same origin), bifid or triple-headed PL (the tendon splits while the muscle belly is intact), and accessory PL [4,5]. Additional variations include the palmaris profundus, where the distal tendon inserts after passing deep to the flexor retinaculum, and reversed PL in which the muscle belly is located distally near the wrist as opposed to the cubital fossa [5]. Collectively, these variations occur in about 9% of the population [5]. Nuanced PL cases are discovered every year, further expanding our knowledge of these variations. For example, reports have found patients exhibiting unique PL variants such as a reversed PL and a three-tendinous head reversed PL a few months prior [6,7]. 

This case report describes the unique presence of PL agenesis in the left upper extremity (LUE) and reversal in the right upper extremity (RUE) of a white male cadaver-donor. We discuss relevant clinical and pathological implications related to PL and its variations. To our knowledge, this is the first case report to identify asymmetric co-occurrence of such PL variations. It is important that variations of the PL are recognized by medical professionals so as to improve patient outcomes during surgical procedures of the anterior forearm.

This article was previously posted to the Research Square preprint server on June 30, 2023.

## Case presentation

**Figure 1:**
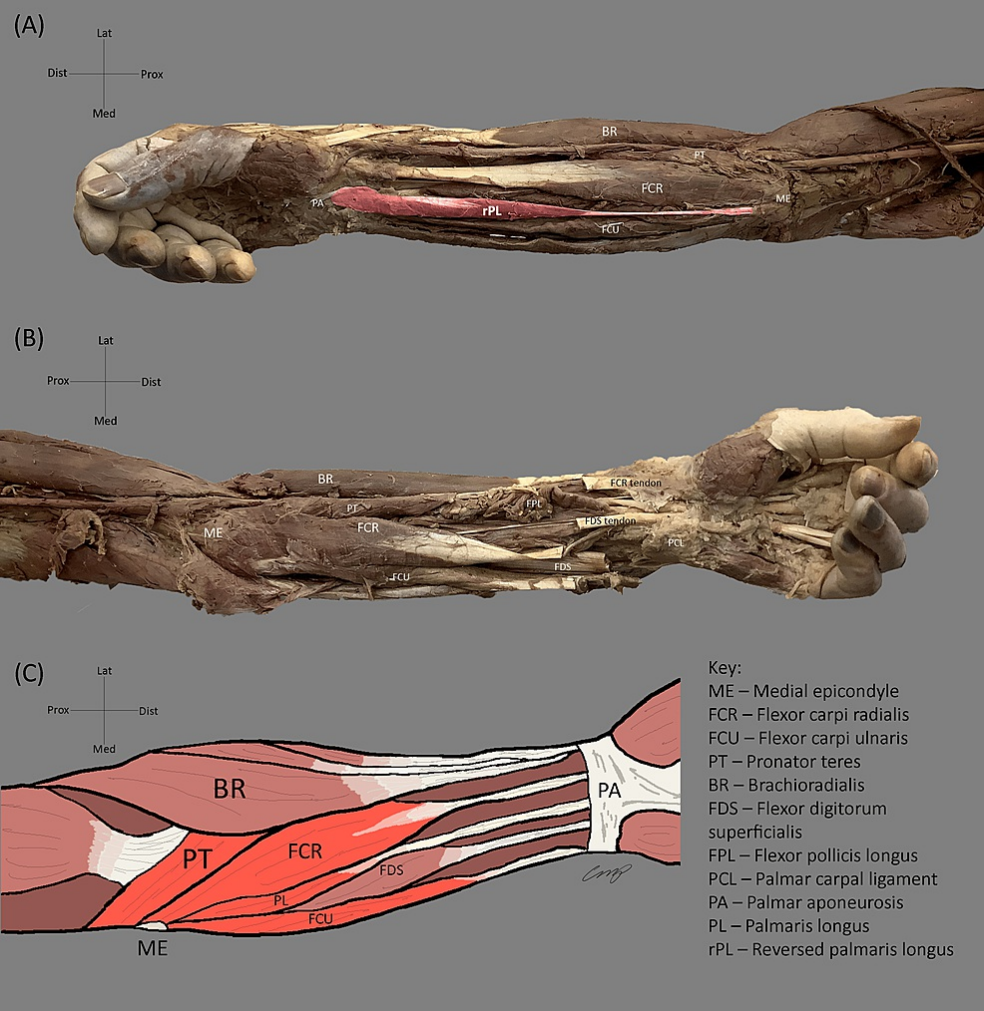
Anatomical variations of the PL muscle in a cadaveric specimen. (A) The forearm of the cadaver-donor's RUE, with reversed PL, is highlighted with the tendinous origin attached to the medial epicondyle of the humerus and the muscle inserting into the PA. (B) The forearm of the cadaver-donor's LUE demonstrates PL agenesis. (C) Schematic of the forearm demonstrating the anatomic relationship of superficial forearm muscles. The PL originates from the medial epicondyle, with the muscle belly running between the FCR and FCU. It inserts on the PA, with the tendon running between tendons of FCR and FDS. PL: palmaris longus; RUE: right upper extremity; PA: palmar aponeurosis; LUE: left upper extremity; FCR: flexor carpi radialis; FCU: flexor carpi ulnaris

Routine anatomical dissection of the bilateral anterior compartment of the forearm on a 79-year-old white male cadaver-donor was performed by first year medical students. The donor gifted his body to the Anatomical Gift Program at The Robert Larner, M.D. College of Medicine. A set of standard dissector instrumentation was used, including probes, scalpels, scissors, and forceps. The superficial flexor muscles of the right forearm were left intact to preserve the observed variation. On the left forearm, the superficial flexor muscles were further dissected in order to observe deeper muscles including the flexor digitorum superficialis and profundus, the flexor pollicis longus, and the pronator quadratus. The anatomical variations on both the left and right PL were photographed using the camera feature on an iPad (Apple, Cupertino, California, United States). Figure 1 was developed using Adobe Photoshop (Version: 21.0.2 20191122.r.57, Adobe, San Jose, California, United States).

Two variations of the PL were identified. On the right, reversal of the PL was observed. Here, the tendon of the right PL was attached at the anatomic origin (medial epicondyle of the humerus) as opposed to the palmar aponeurosis in the wrist (Figure 1). The muscle belly of the right PL was attached at the anatomic insertion (palmar aponeurosis) as opposed to the medial epicondyle. On the left, complete PL agenesis was noted as indicated by Figure 1.

## Discussion

This case documents the first presentation of an individual with two concurrent PL variations. In particular, the cadaver-donor demonstrated PL agenesis on the left and reversal on the right. These variations are well-documented in the literature which indicate PL reversal is more common in the LUE and agenesis incidence rates range from 1.5% to 63.9% [2-4]. 

The PL and its associated variants may have evolved as a result of specialized movement such as bipedal locomotion or tool usage [8]. In this study, the authors hypothesize that the PL in humans is undergoing regression, evidenced by the smaller belly of this muscle in comparison to other muscles of the forearm [8]. These evolutionary pressures varied widely, translating to significant variation within the PL [3,8]. This includes its complete absence in certain species [3]. A recent report by Vučinić et al. looked at the presence of the PL in 300 identical twins, identifying the absence of the PL in one set of twins [9]. The authors speculate that the muscle is inherited in an autosomal dominant fashion and that the lack of the muscle might be tied to a single dominant gene with variable expression [9]. 

There are many recorded variations in the PL muscle bellies including bifurcation, trifurcation, and fusion (defined as the merging of the PL and FCU tendons) [10]. Additional variations have been recorded that denote the agenesis and reversal of the PL [5,11-13]. Generally, agenesis or reversal of the PL has been observed unilaterally or bilaterally [2,5,13-15]. In regard to bilateral documentations, reports documented symmetric changes in the PL [14,16]. However, in our case, we observed the first asymmetric change in which there were reversal and agenesis of the PL in the same cadaver-donor. 

Clinically, PL variations have been known to cause neuropathies as a result of neurovascular compression in the carpal tunnel or Guyon's canal. In particular, PL reversal has been shown to present with swelling, numbness, and pain that is exacerbated with wrist flexion [17]. This exacerbation can be attributed to the hypertrophy associated with repetitive flexion, producing effort-related compartment syndrome. Ultimately, the enlargement of the muscle belly can result in nerve and muscle damage through ischemia [11,17]. Conversely, PL agenesis is classified as a clinically benign condition. Note that the PL is not essential for normal forearm function [18]. In fact, its absence does not lead to any significant decrease in grip strength. This quality is advantageous in surgical procedures, allowing the muscle to be harvested for tendon graft surgeries. 

When identifying PL abnormalities, testing is required especially in the context of wrist pain syndromes and for procedure planning like in tendon transfer [19]. Initial PL identification tests include Schaeffer's test in which the patient is asked to oppose the thumb and fifth finger. The PL should appear as a slender tendon in the wrist; however, if the tendon is not observed, further testing is required to confirm the presence of potential PL variations. Such tests include Thompson's test (the patient makes a fist, flexes the wrist, and opposes thumb over fingers), Mishra's test I (the examiner hyperextends the metacarpophalangeal joints of all fingers, while the patient is asked to flex wrist against resistance), Mishra's test II (the patient abducts thumb against resistance with wrist in flexion), and Pushpakumar's two-finger sign test (the second and third fingers are extended, while the wrist and fourth to fifth fingers are flexed, and the patient is asked to flex and oppose the thumb) [4,19,20]. Additional radiographic studies such as ultrasonography and cross-sectional MRI may be necessary to confirm the contribution of PL abnormalities to pathology [17]. Importantly, these tests and radiographic studies must be done bilaterally. Given the asymptomatic presentation of PL agenesis, a patient may not report any anomalies on one side while experiencing symptoms like neurovascular compression in the carpal tunnel on the other [18]. Overlooking the muscle's bilaterality may exacerbate a patient's condition. In the context of our cadaver-donor, such due diligence would have been especially critical considering the patient presented with both reversal and agenesis of the PL. These qualities may be overlooked considering the abnormalities in the PL reported in the literature thus far have been unilateral or symmetrical. 

## Conclusions

By presenting a distinct case of asymmetrical variation, we aim to raise physician awareness of the diversity that can possibly be demonstrated in the PL. This awareness can increase clinical diagnostic accuracy and alleviate potential stress for the patient especially in the context of abnormal wrist pain or tendon grafting. Given the wide range of anatomical variations present in the PL, meticulous tests must be administered in order to recognize anatomical abnormalities. Our hope is that through ample case documentation, clinical science can improve currently utilized diagnostic tools to ensure the best possible outcomes for patients, even when considering uncommon anatomical variants. 
